# Increase in Anxiety-Related Out-of-Hours Primary Care Consultations Since COVID-19: An Observational Study Using Routine Data in Flanders

**DOI:** 10.3389/frhs.2021.763739

**Published:** 2021-11-16

**Authors:** Kris Van den Broeck, Stefan Morreel, Inge Glazemakers, Veronique Verhoeven, Eva Rens, Hilde Philips

**Affiliations:** ^1^Family Medicine and Population Health (FAMPOP), University of Antwerp, Antwerp, Belgium; ^2^Collaborative Antwerp Psychiatric Research Institute (CAPRI), University of Antwerp, Antwerp, Belgium

**Keywords:** mental health, anxiety, out-of-hours medical care, organization of care, primary care

## Abstract

**Background:** Survey studies suggest that COVID-19 has had a negative impact on the population's mental well-being. Routine registration data allow a more objective way for investigating such associations, complementary to self-report measures. This study investigates the level of out of hours (OOH) consultations for psychological problems since the start of the COVID-19 pandemic in Flanders, Belgium.

**Methods:** The iCAREdata database is a clinical research database with routine data of OOH care, covering a large area in Flanders, Belgium. After defining the first wave and the second wave of COVID-19 in Flanders in time, we compared the number of consultations regarding psychological problems (in general, anxiety-related, depression-related, and sleep-related) between those periods, the period in between these waves, and the period before the start of COVID-19.

**Results:** A significant rise in OOH consultations due to psychological—and more specifically, anxiety-related—problems is observed since the start of the COVID-19 pandemic in Flanders. Elevated levels are observed until the second wave. This finding is in sharp contrast with the general pattern of lower demand for primary healthcare during this period. The majority of these supplementary consultations happened by phone. Consultations regarding depression-related problems did not change over time. Sleep disturbances in the OOH setting were more common after the first wave.

**Conclusion:** Despite some limitations, a large Flemish database with routine data on OOH care shows an increase in the number of consultations regarding psychological problems in general and anxiety-related problems since the start of the COVID-19-pandemic until the second wave.

## Key Points

- According to routine registrations of out of hours care, consultations due to psychological problems in general and anxiety problems in particular were more common during the COVID-19 pandemic in Flanders, Belgium.
- According to routine registrations of out of hour care, consultations due to depression-related problems did not change over time during the COVID-19 pandemic in Flanders, Belgium.
- According to routine registrations of out of hour care, consultations due to sleep disturbances were more common in later phases of the COVID-19 pandemic in Flanders, Belgium.
- The use of routine registration data may be complementary to self-report measures to detect trends in the population's mental well-being—which is important to react accordingly—in critical times, e.g., during a pandemic.

## Introduction

Many survey studies have shown that COVID-19 and the measures to control the pandemic have (had) a strong and negative impact on the population's mental well-being (1–3). Moreover, it has been suggested that fluctuations in mental well-being correspond with the strengthening and the relaxation of the measures, which is generally in accordance with the severity of the pandemic locally (3, 4). Previous research concerning the first COVID-19 wave in Belgium revealed an increase in diagnoses related to mental health and a decrease in the demand for primary care consultations unrelated to COVID-19 (5).

In contrast to survey-based research, in which often voluntary respondents are questioned about their well-being, routine registration data allow us to more objectively study the prevalence of (mental) health problems on a regular basis in a large share of citizens, and how fluctuations in health problems are related to the evolution of the COVID-19 pandemic. The aim of this study is to describe the demand for care regarding mental health problems during out of hours (OOH) care before and during the COVID-19 pandemic in Flanders, Belgium.

The first wave of COVID-19 in Belgium began at the beginning of March 2020, peaked on March 28th (with 642 admissions daily) and ended by May 4th. During that period, Belgium went in lockdown. Non-essential businesses were closed since March 18th, and citizens were requested to work from home and to limit interpersonal contacts. Non-urgent medical appointments were adjourned and patients showing symptoms of COVID-19 were asked to contact their GP by phone. Triage stations were set up. Though citizens were still requested to limit their contacts and to work from home if possible, schools reopened and social life was gradually resumed by the beginning of May, as was non-urgent care. Figures dropped in summer but rose again at the end of September—a second wave began, following an exponential curve from October 10th. Again, more stringent measures were implemented. Restaurants and professional salons were closed, and non-essential shops could only be visited on request or to take out pre-ordered objects. Schools, however, did not close, although pupils of 14 years and older and students were obliged to follow (a part of) their lessons online. The autumn holidays were extended with an additional week, after which the number of infections declined again, but the second wave was followed by a high plateau phase.

We hypothesize that the severity of the COVID-19 pandemic in Flanders is reflected in the number of patients that visit a practitioner for psychological problems OOH. More specifically, we expect that routine registrations referring to psychological problems in general, and to anxiety-, depression-, and sleep-related problems in particular, will be more prevalent in the registry during the two COVID-19 waves, compared to the period before COVID-19 and the period in between the waves.

## Methods

### Data Collection

The iCAREdata database (Improving Care And Research Electronic Data Trust Antwerp) is a central, clinical research database on OOH care that is provided to a large population of inhabitants (all ages) in Flanders, Belgium (6, 7). Every weekend, routine data of general practitioner (GP) cooperatives are added to the database, respecting privacy regulations and ethical considerations. In 2019, the area covered by the participating health care professionals / units counted 1.914.541 inhabitants; additional partnerships were added in 2020, resulting in an area that covers 3.162.345 inhabitants (48% of the Flemish population, representative for the entire Flemish population).

All consultations during weekends and bank holidays (further referred to as weekends) in 2019 and 2020 were included. Patients without a Belgian national insurance number were excluded. The exact number of exclusions is unknown but below five percent. GPCs need this number for billing to the obliged national health insurance so they will obtain this number whenever possible. The weekend of 18/09/2020 was excluded because of a data collection problem.

The receptionists of the GPCs registered the type of contact (telephone contact, physical contact or home visit). GPs had to register a diagnosis by selecting a single clinical label out of a Belgian list which is linked to the International Classification of Primary Care, 2nd edition (ICPC-2). Due to software limitations, it was not possible to register several diagnoses. GPs were asked to use specific codes for consultations regarding a proven or suspected case of COVID-19, a close contact of a proven case, for COVID-19 testing only and for fear of COVID-19 (which should be interpreted as questions about the disease, concerns about being infected, … but not as anxiety or phobia). All these COVID-19-related consultations were excluded from the analyses. In case a patient had multiple consultations within a 6-hour timeframe, only the first physical consultation was studied if available, else the first telephone consultation was studied.

Following the evolution of COVID-19 and its associated measures in Flanders, we defined the “Pre-COVID-19” era (69 weekends and holidays; between the start of 2019 and until March 9th 2020), the “First wave” (7 weekends; between March 13th and April 27th 2020), the “Second wave” (9 weekends; between October 9th and November 30th, 2020), and the period between and after the waves (29 weekends; between May 1st and October 8th 2020 and between December 4th and December 31st 2020).

### Analysis

The following variables were included in the analyses: type of contact (home visit, physical consultation or telephone consultation), timing (day, hours and minutes), age (years), sex (male or female), and diagnosis (ICPC-2 coded).

Patients with a COVID-19 specific diagnosis were excluded. We computed the number of patients requesting OOH care for any non-COVID-19 problem and for psychological problems (all P-scores of ICPC-2) per 100.000 inhabitants per weekend for each of the defined periods, and compared them using ANOVA. If significant, Fisher's Least Significant Difference (LSD) *post-hoc* tests were used to explore the differences between the time periods. We repeated this procedure for anxiety (P01 + P74 + P79, with P01 = “feeling anxious/nervous/tense”; P74 = “anxiety disorder/anxiety state”; P79 = “phobia/compulsive disorder”), depression (P03 + P76, with P03 = “feeling depressed”; P76 = “depressive disorder”) and sleep disturbances (P06), to investigate which issues in particular were affected. The incidences were plotted for illustration. All data were analyzed using JMP Pro 15.

Ethical approval for this study was obtained from the ethics committee of Antwerp University Hospital (approval number B3002020000058).

## Results

### General Overview

**Figure 1:**
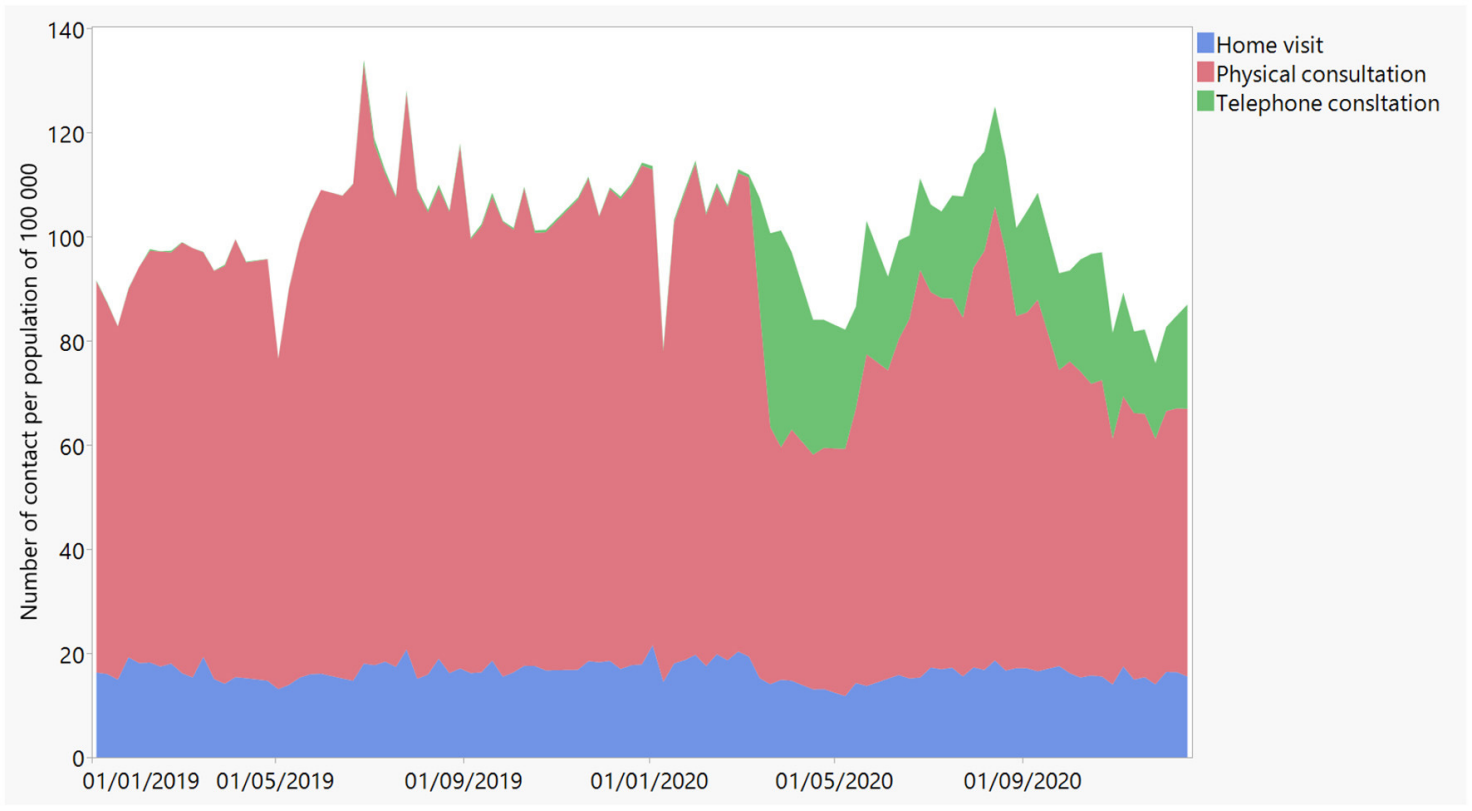
Evolution of all physical consultations (in red), consultations by phone (in green), and home visits (in blue) over time in 2019 and 2020 as registered by iCAREdata, regardless the diagnosis.

The database contains 357.710 eligible consultations. The mean age of the consulting patients is 34 years (*SD* = 23) and 54.82% is female. Figure 1 shows the evolution of all OOH consultations over time per 100.000 inhabitants in 2019 and 2020, divided by type of contact. At the beginning of the pandemic, OOH care dropped dramatically. Especially physical consultations declined, but were partly compensated by the increase in consultations by phone, which were recommended during the pandemic. The sum of physical consultations, telephone consultations and home visits shows a net decrease in non-COVID-19 related healthcare seeking behavior.

### The Prevalence of OOH Encounters Due to Psychological Problems During COVID-Times

**Figure 2:**
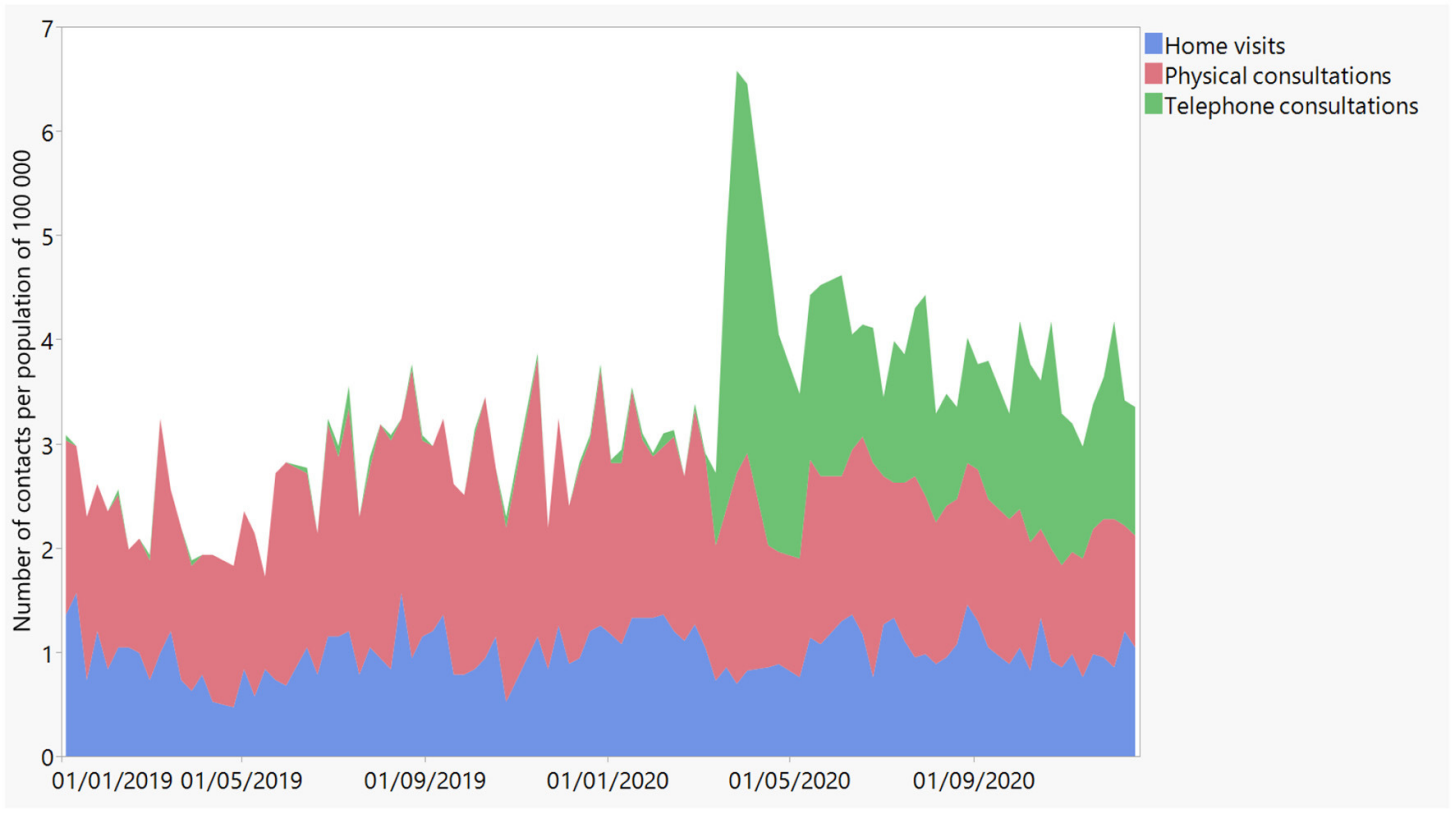
Evolution of physical consultations (in red), consultations by phone (in green), and home visits (in blue) for psychological reasons (i.e., all P-codes of ICPC-2) over time in 2019 and 2020 as registered by iCAREdata.

A total of 8.990 consultations (2.52% of all consultations; 95% CI 2.47–2.57) were coded for psychological diagnoses (ICPC-2 chapter P). Figure 2 displays the sum of OOH home visits, physical consultations and telephone consultations due to psychological problems (all ICPC-2 P-codes) per 100.000 inhabitants over time in 2019 and 2020. Remarkably, about half of all contacts due to psychological problems (52%) during the first wave were by phone. In between the two waves, 36% of contacts due to psychological problems were by phone.

The descriptive statistics of the level of consultations before and during COVID-19 are reported in Table 1. The demand for care regarding psychological problems increased from 2.69/100.000 before COVID-19 to 5.24/100.000 during the first wave, and dropped again to 3.77/100.000 and 3.27/100.000 in between waves 1 and 2 and during the second wave, respectively. This evolution was significant, *F*(3, 111) =17.03, *p* < 0.0001. Fisher's LSD *post-hoc* analysis was used to investigate the size and significance of the differences between the different time frames. The results of the *post-hoc* tests are presented in Table 2. No significant difference was found between the number of contacts in between waves 1 and 2 and those registered during wave 2, nor between the number of contacts during the pre-COVID-19 era and those registered in wave 2. However, all other differences in contact volume do differ significantly with *t* = 1.98, *p* < 0.05 or less.

**Table 1:** Descriptive statistics of the levels of consultations due to psychological problems in general, anxiety-related problems, depression-related problems and sleep-related problems before COVID, during the first and second wave, and between the waves.

	**Period**	**Mean incidence per 100.000**	**SD**	**95% CI**
All psychological consultations	Pre-COVID	2.69	0.86	2.48–2.89
	First wave	5.24	1.55	3.81–6.67
	Between waves	3.77	1.37	3.26–4.28
	Second wave	3.27	0.78	2.67–3.87
Anxiety-related consultations	Pre-COVID	0.62	0.25	0.55–0.68
	First wave	2.37	0.94	1.50–3.25
	Between waves	1.14	0.39	0.99–1.29
	Second wave	1.01	0.35	0.74–1.28
Depression-related consultations	Pre-COVID	0.19	0.12	0.17–0.22
	First wave	0.23	0.13	0.11–0.35
	Between waves	0.23	0.11	0.19–0.27
	Second wave	0.19	0.10	0.12–0.27
Sleep-related consultations	Pre-COVID	0.12	0.08	0.10–0.14
	First wave	0.14	0.04	0.10–0.18
	Between waves	0.16	0.10	0.12–0.20
	Second wave	0.07	0.05	0.04–0.11

**Table 2:** Results of the pairwise comparisons of the *post-hoc* analysis using the Fisher's Least Significant Difference (LSD) test.

	**Comparison**	**Difference**
General	Pre-COVID—First wave	−2.55^***^
	Pre-COVID—Between waves	−1.08^***^
	Pre-COVID—Second wave	−0.58
	First wave—Between waves	1.47^*^
	First wave—Second wave	1.97^**^
	Between waves—Second wave	0.50
Anxiety-related	Pre-COVID—First wave	−1.75^***^
	Pre-COVID—Between waves	−0.52^***^
	Pre-COVID—Second wave	−0.39^*^
	First wave—Between waves	1.23^***^
	First wave—Second wave	1.36^***^
	Between waves—Second wave	0.13
Sleep-related	Pre-COVID—First wave	0.02
	Pre-COVID—Between waves	0.04^*^
	Pre-COVID—Second wave	0.04
	First wave—Between waves	0.02
	First wave—Second wave	0.07
	Between waves—Second wave	0.08^*^

*** *p < 0.0001*;

** *p < 0.0005*;

* *p < 0.0050*.

### The Pre-valence of OOH Encounters Due to Anxiety- or Depression-Related Problems and Sleep Disturbances

It was further explored what kind of psychological problems added to the observed pattern. To investigate the evolution of anxiety-related problems, all consultations which were registered with P01, P74, or P79 were taken into account. Whereas before COVID-19, anxiety problems accounted for 23.19% of all psychological problems, this share rose to 45.27% during the first wave. After that first wave, still about one third of all psychological problems are anxiety-related.

The evolution of anxiety is similar to that of psychological problems in general (Table 1). Significant changes were observed between the time periods, *F*(3,110) = 55.31, *p* < 0.0001. A rise from 0.62/100.000 consultations for anxiety-related problems before COVID-19 to 2.37/100.000 consultations during the first wave was observed. This figure decreased to 1.14/100.000 and 1.00/100.000 in between waves and during the second wave, respectively. The results of the *post-hoc* analysis are presented in Table 2. All differences between the time periods are significant with *t* = 1.98 and *p* < 0.005 or less, except the difference of anxiety-related contacts in between the waves and in the second wave.

To investigate depression-related problems, all consultations that were coded with P03 or P76 were taken into account. Remarkably, before COVID-19, depression accounts for 7.22% of all OOH consultations on psychological problems. During the first wave, only 4.39% of the consultations on psychological distress were related to depression. No significant differences were observed when comparing the prevalence rates of depression related consultations between the different periods in the light of the COVID-pandemic, *F*(3,110) = 0.71, n.s.

Finally, we investigated sleep disturbances (P06). Before COVID-19, sleep disturbances accounted for 4.35% of all OOH consultations on psychological problems. During the first wave, 2.67% of all consultations on mental problems were on sleep problems. Analyses revealed significant differences regarding the demands on sleep disturbances in OOH over time, *F*(3, 110) = 2.80, *p* < 0.05. Especially the period in between waves seemed critical, as the number of request in this period was significantly higher compared to both the period before COVID-19 and the second wave, with *t* = 1.98, *p* < 0.05. No further differences were found.

## Discussion

To our knowledge, this study is the first that explored the prevalence and evolution of psychological problems against the background of the COVID-19-pandemic using a large database containing systematically collected clinical routine data of OOH care, including the diagnoses. A first finding is that OOH consultations in general decreased during the pandemic, while consultations specifically for psychological problems increased. The number of physical consultations also decreased, while telephone contacts increased. These general trends were described by Morreel et al. (5).

Our analyses revealed a significant rise in consultations due to psychological problems in general, and in anxiety-related problems in particular, since the start of the COVID-19-pandemic in Flanders. A peak during the first wave is observed. Although there was no (significant) rise during the second wave compared to the period in between waves, the demand for OOH care regarding anxiety-related problems remained elevated after the first wave compared to the period before COVID-19. Furthermore, consultations for sleep disturbances were more common in the weeks between the two waves compared to the pre-COVID-19 era or the second wave. In contrast, depression-related problems did not show any fluctuations over time.

These findings contrast with the general pattern of a decline in healthcare seeking behavior for primary and acute care during this phase of the pandemic, in Belgium as in the rest of the world (5, 8–10). While contacts for general health or emergency care declined, the number of OOH contacts for mental health problems rose. This trend was also found for emergency department visits in the US, where various mental health problems as well as social problems (e.g., overdoses) were higher in March through October 2020 as compared to the same period in 2019 (11). Interestingly, a study on primary care-recorded mental health problems suggested that the incidence of primary care- recorded depression and anxiety first declined significantly in English general practices, but returned to expected levels in September (12). These findings suggest that the decrease in regular mental health care reflects missed opportunities for care, and is compensated by the increased use of OOH care and emergency care. Missed care opportunities ultimately lead to an increase of the severity and the accumulation of mental health needs. The increased use of OOH care for mental health reasons can therefore also be seen as an indication of unmet mental health needs in the population.

Anxiety-related problems, including panic reactions, very well-fit the situation of the COVID-19 pandemic. A new and unknown virus obviously evokes a lot of stress responses. While some people may be afraid of getting ill or losing a loved one due to COVID-19, other factors such as financial hardship or fear of losing one's job may also play a role. Measures inducing social isolation, however, would be more likely to evoke feelings of loneliness or depression. Yet, regarding anxiety, social isolation may limit one's coping opportunities to effectively deal with anxiety as well-potentially enlarging the demands for help for anxiety-related problems. In regular (i.e., not OOH) primary care, the most common psychological diagnoses in Flanders are depression, acute stress, sleeping disturbances and “feeling anxious”, respectively (13). The finding that depression and sleeping problems are less common in OOH care compared to non-OOH care while anxiety is more often a main theme in OOH care, may also mean that anxiety feels more urgent and pressing to patients, causing them to contact a physician outside the regular hours.

Our results show that, although possible mental health issues were addressed at times in the media in Belgium (messages focussing on staying fit, going outside for a walk, …), this was not enough to compensate for the multiple stresses communities experience during the pandemic. Moreover, regular registration systems nor surveys might not be able to capture all problems. Therefore, governments need to think about advanced mental health infrastructures and systems that address well-being on a population level to better deal with these problems (14, 15).

One of the limitations of this study is that practitioners are unable to register more than one reason for encounter per consultation. This leads to underregistration when the patient also presents with a physical complaint. As psychological problems may be present as co-morbid problems next to a more prominent physical question during a consultation, our data might be underestimations of reality. Also, it should be noted that the data we used are collected on OOH care. This means that practitioners see patients they are not familiar with. Complex problems, like depressive state, might be more difficult to diagnose by doctors that do not know the patient when the patient does not clearly outline his emotional state. Additional data on regular care, delivered by familiar GP's, might add to the picture. It should be noted that, although our database covers a large area, the number of consultations regarding psychological problems are rather limited. Patients without a national insurance number were excluded so we do not have data on this vulnerable population. Finally, the findings should be carefully translated to other contexts, in particular to other countries where other forms of OOH care may be used.

Nevertheless, despite these limitations, our study shows the importance of a complementary use of routine registrations for managing (mental) health crises for policy makers and the organization of care.

## Data Availability Statement

The data analyzed in this study is subject to the following licenses/restrictions: The iCAREdata dataset is used. Researchers can consult the database after applying for access. Due to privacy rules, iCAREdata is only allowed to provide aggregated data. Requests to access these datasets should be directed to https://www.uantwerpen.be/en/projects/icaredata/icaredata-project.

## Ethics Statement

Ethical approval for this study was obtained from the ethics committee of Antwerp University Hospital (Approval No. B3002020000058). Written informed consent for participation was not required for this study in accordance with the national legislation and the institutional requirements.

## Author Contributions

KVdB, SM, IG, VV, ER, and HP equally developed the research question and the research plan. SM prepared the data and conducted the analyses. KVdB wrote the different drafts of the manuscript. All other authors commented the drafts and added equally to an improvement of the manuscript.

## Conflict of Interest

The authors declare that the research was conducted in the absence of any commercial or financial relationships that could be construed as a potential conflict of interest.

## Publisher's Note

All claims expressed in this article are solely those of the authors and do not necessarily represent those of their affiliated organizations, or those of the publisher, the editors and the reviewers. Any product that may be evaluated in this article, or claim that may be made by its manufacturer, is not guaranteed or endorsed by the publisher.
